# LncRNA LOC653786 promotes growth of RCC cells via upregulating FOXM1

**DOI:** 10.18632/oncotarget.24027

**Published:** 2018-01-08

**Authors:** Fan Yang, Qingjian Wu, Yan Zhang, Haojun Xiong, Xinzhe Li, Bo Li, Wei Xie, Le Zhang, Min Xu, Kebin Zhang, Fengtian He

**Affiliations:** ^1^ Department of Biochemistry and Molecular Biology, College of Basic Medical Sciences, Third Military Medical University, Chongqing 400038, China; ^2^ Central Laboratory, Xinqiao Hospital, Third Military Medical University, Chongqing 400037, China; ^3^ Department of Urology, Xinqiao Hospital, Third Military Medical University, Chongqing 400037, China; ^4^ Center for Disease Control and Prevention, Chengdu Military Region, Chengdu 610021, China

**Keywords:** LOC653786, FOXM1, long noncoding RNA, renal cell carcinoma, cell growth

## Abstract

Renal cell carcinoma (RCC) is the most common kidney malignancy with poor prognosis. Recently, long noncoding RNAs (lncRNAs) have been demonstrated as important regulators in multiple cancers including RCC. LOC653786 is a lncRNA, but its role in cancer remains unclear. In this study, we for the first time found that LOC653786 was upregulated in RCC tissues and cell lines, and this lncRNA promoted growth and cell cycle progression of RCC cells. Moreover, we showed that LOC653786 elevated the expression of forkhead box M1 (FOXM1) and its downstream target genes cyclin D1 and cyclin B1 in RCC cells. Reporter assay revealed that LOC653786 enhanced the transcriptional activity of *FOXM1* gene promoter. Additionally, knockdown of FOXM1 attenuated the LOC653786-enhanced growth and cell cycle progression of RCC cells. Meanwhile, silencing of LOC653786 suppressed RCC cell growth and cell cycle progression, which was alleviated by overexpression of FOXM1. The *in vivo* experiments in nude mice showed knockdown of LOC653786 repressed xenograft tumor growth and FOXM1 expression. In conclusion, our results demonstrate that LOC653786 accelerates growth and cell cycle progression of RCC cells via upregulating FOXM1, suggesting that the ‘LOC653786/FOXM1’ pathway may serve as a novel target for RCC treatment.

## INTRODUCTION

Renal cell carcinoma (RCC) is the most common malignancy of kidney which accounts for approximate 2%-3% of human malignant neoplasms [1], and its incidence and mortality are rising these years [2]. Clear cell renal carcinoma (ccRCC) represents the most common RCC, which occupies about 75%-80% of RCC [1]. Moreover, the treatment of RCC remains a big challenge at present because of its poorly responding to chemotherapy and radiotherapy [3, 4]. There has been little progress in early diagnosis and treatment of RCC despite persistent efforts in the past several decades [4–6]. Therefore, it is urgent to look for early diagnosis biomarkers and therapeutic targets.

Long noncoding RNAs (lncRNAs) are a subgroup of transcripts with more than 200 nucleotides and limited coding potential [7]. It has been reported that lncRNAs can regulate gene expression at multiple levels, including chromatin modification, transcription and post-transcriptional processing [8–11]. Furthermore, mounting evidences have revealed that lncRNAs play critical roles in a number of cancers by affecting many cellular processes, such as cell cycle, survival, migration, metabolism and autophagy [12–16]. Recently, some lncRNAs (such as MALAT1, HOTAIR, LncARSR, etc.) have shown importance in RCC [17–22]. However, it remains largely unknown about the profiles of lncRNAs in RCC.

LOC653786 is a lncRNA, consist of 2661 nucleotides and located in chr16p12.2 [23]. So far it is unclear whether LOC653786 is associated with RCC. In this study, we found that LOC653786 was elevated in RCC tissues and cell lines, and this lncRNA promoted growth and cell cycle progression of RCC cells via upregulating forkhead box M1 (FOXM1). The *in vivo* experiments in nude mice showed that knockdown of LOC653786 dramatically repressed xenograft tumor growth and FOXM1 expression. These results indicate that the pathway ‘LOC653786/FOXM1’ accelerates RCC cell growth, suggesting that this pathway may serve as a novel target for the treatment of RCC.

## RESULTS

### LOC653786 is upregulated in RCC tissues and cell lines

**Figure 1:**
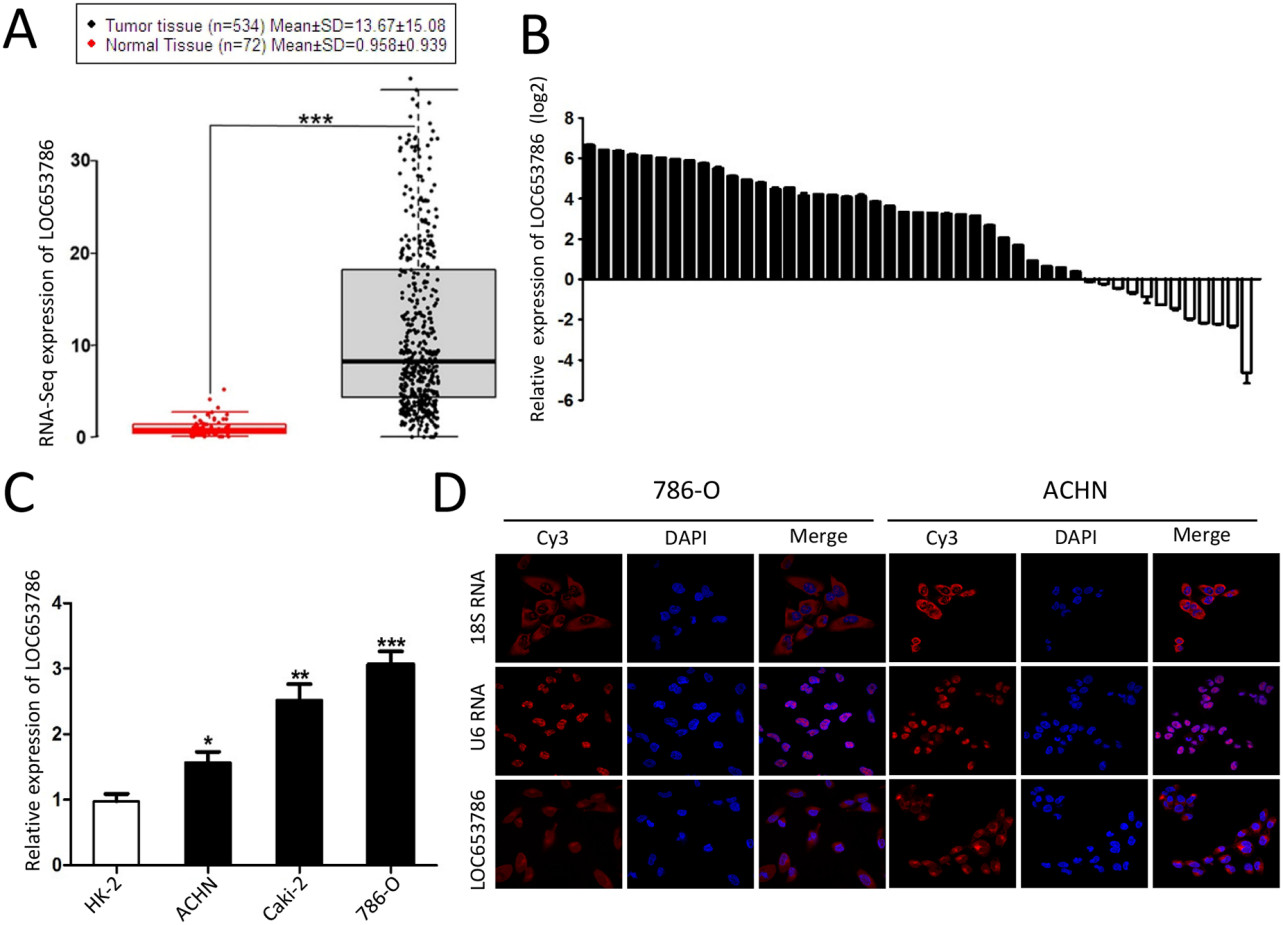
LOC653786 is upregulated in RCC tissues and cell lines **(A)** Analysis of the expression of LOC653786 in ccRCC tissues and the normal tissues in TCGA datasets. **(B)** The expression of LOC653786 in 50 pair-wise ccRCC tissues and the corresponding adjacent non-tumor tissues was assayed by qPCR. **(C)** The expression of LOC653786 in RCC cell lines (ACHN, Caki-1 and 786-O) and the relative normal proximal tubule epithelial cell line HK-2 was determined by qPCR. **(D)** FISH assay was performed to detect the distribution of LOC653786 in RCC cells, taking 18S RNA as a cytoplasmic RNA control and U6 RNA as a nuclear RNA control. The lncRNA probe mix and control RNA probe mix were separately labeled with Cy3, and the nuclei were counterstained with DAPI. The high resolution images were captured with a laser scanning confocal microscope. ^*^P<0.05, ^**^P <0.01, ^***^P <0.001.

As shown in Figures 1A and Supplementary Figure 1, analysis of the Cancer Genome Atlas (TCGA) datasets showed that LOC653786 was upregulated in RCC tissues compared to normal tissues (Figure 1A), and the level of LOC653786 in RCC tissues was much higher than that in normal tissues in different histological grades (Supplementery Figure 1A) and TNM stages (Supplementery Figure 1B). Subsequently, the expression of LOC653786 in 50 pair-wise RCC tissues and the corresponding adjacent non-tumor tissues were detected. As shown in Figure 1B, LOC653786 was elevated in 76% (38 of 50) of RCC tissues. Meanwhile, the level of LOC653786 in RCC cell lines (ACHN, Caki-1 and 786-O) was higher compared to the relative normal proximal tubule epithelial cell line HK-2 (Figure 1C). Moreover, RNA fluorescence in situ hybridization (FISH) showed that the majority of LOC653786 distributed in cytoplasm, and the minority of LOC653786 distributed in nucleus (Figure 1D). Taken together, the above results demonstrate that RCC tissues and cell lines have a higher level of LOC653786, suggesting that LOC653786 may play an important role in RCC development and progression, and this lncRNA may serve as a novel potential biomarker and therapeutic target of RCC.

### LOC653786 promotes RCC cell growth

**Figure 2:**
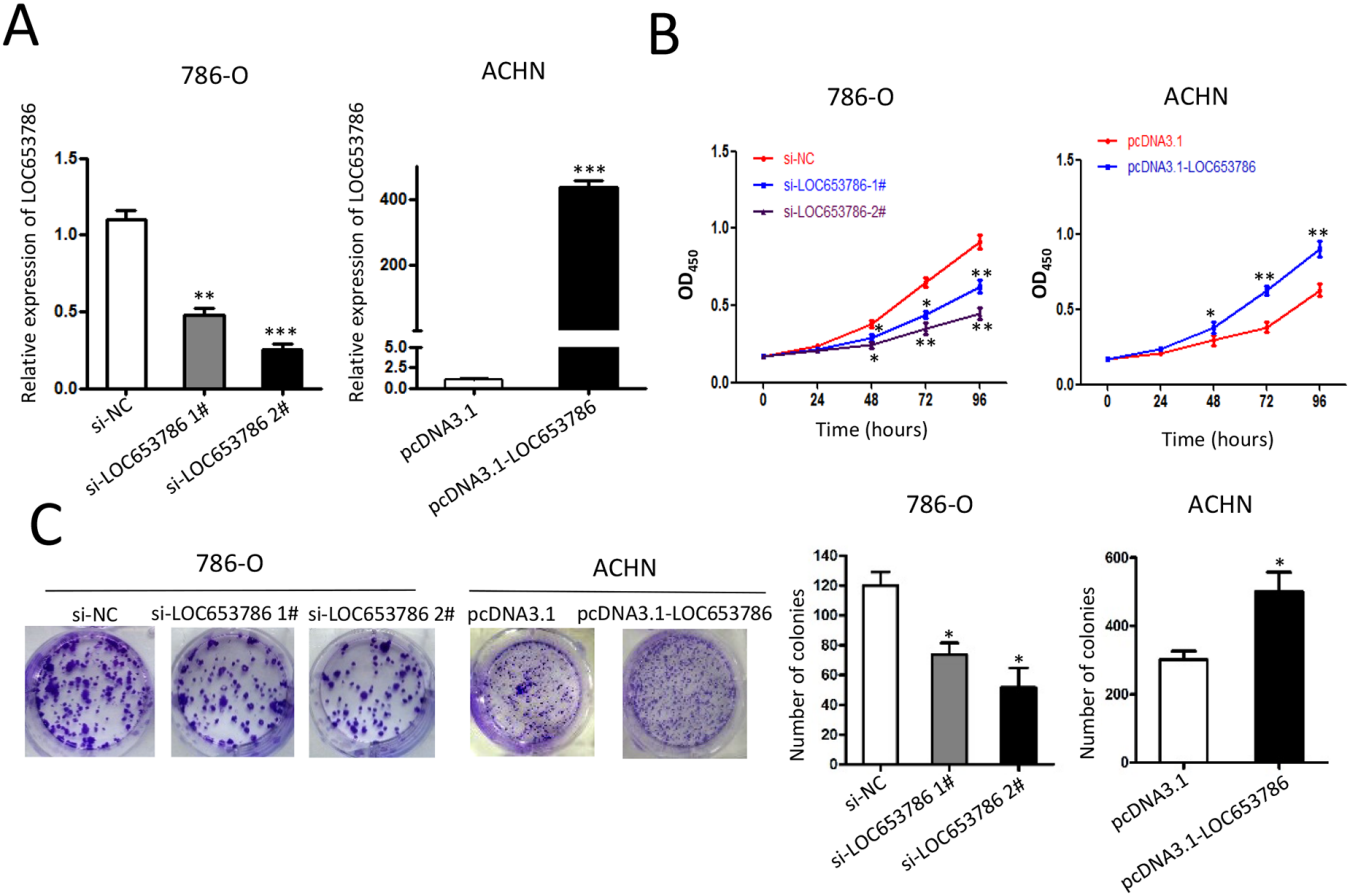
LOC653786 promotes RCC cell growth 786-O and ACHN cells were separately transfected with the indicated siRNAs and expression plasmids for 48 h, then the level of LOC653786 was examined by qPCR **(A)**, the cell viability was determined by CCK-8 assay**(B)**, and the colony-forming ability was detected by colony formation assay **(C).** ^*^P<0.05, ^**^P<0.01, ^***^P<0.001.

As shown in Figure 2, CCK-8 and colony formation assays showed that knockdown of LOC653786 by siRNA significantly suppressed the survival and colony formation of RCC cells, while overexpression of LOC653786 dramatically increased RCC cell viability and colony formation. These results indicate that LOC653786 promotes growth of RCC cells *in vitro*.

### LOC653786 accelerates RCC cell cycle progression

**Figure 3:**
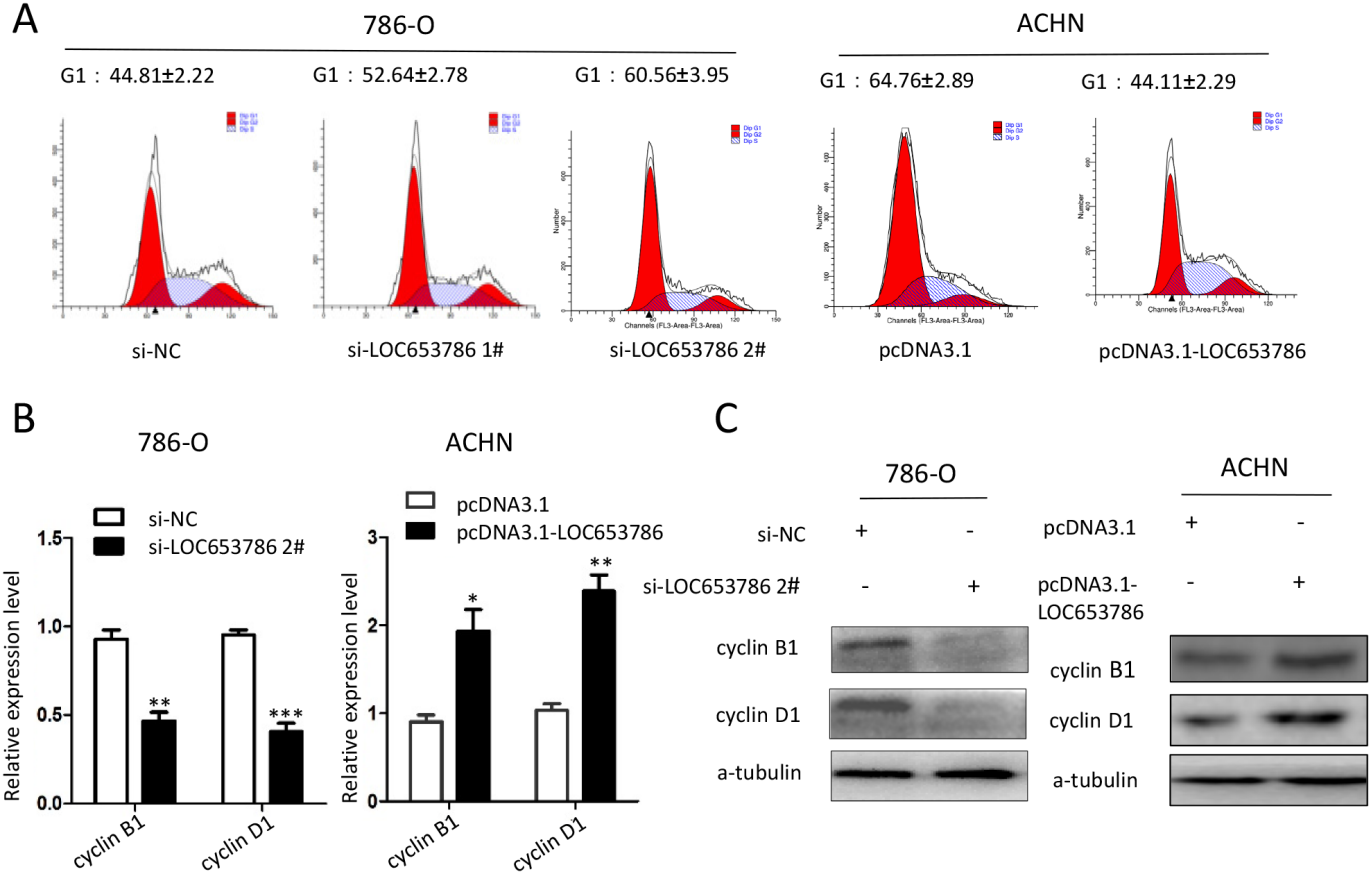
LOC653786 accelerates cell cycle progression of RCC cells 786-O and ACHN cells were separately transfected with the indicated siRNAs and expression plasmids for 48 h, then the cell cycle was analyzed with flow cytometry **(A)**, and the mRNA and protein levels of cyclin D1 and cyclin B1 were detected by qPCR **(B)** and Western blot **(C)**. ^*^P<0.05, ^**^P<0.01, ^***^P<0.001.

Flow cytometry analysis revealed that knockdown of LOC653786 in RCC cells led to a cell cycle arrest in G1 phase, while overexpression of LOC653786 accelerated G1 progression in RCC cells (Figure 3A). The quantitative real-time PCR (qPCR) and Western blot assays showed that LOC653786 induced the expression of cyclin B1 and cyclin D1 at both mRNA and protein levels (Figure 3B and 3C). These data indicate that LOC653786 promotes RCC cell cycle progression.

### LOC653786 upregulates FOXM1 by enhancing its promoter transcriptional activity

**Figure 4:**
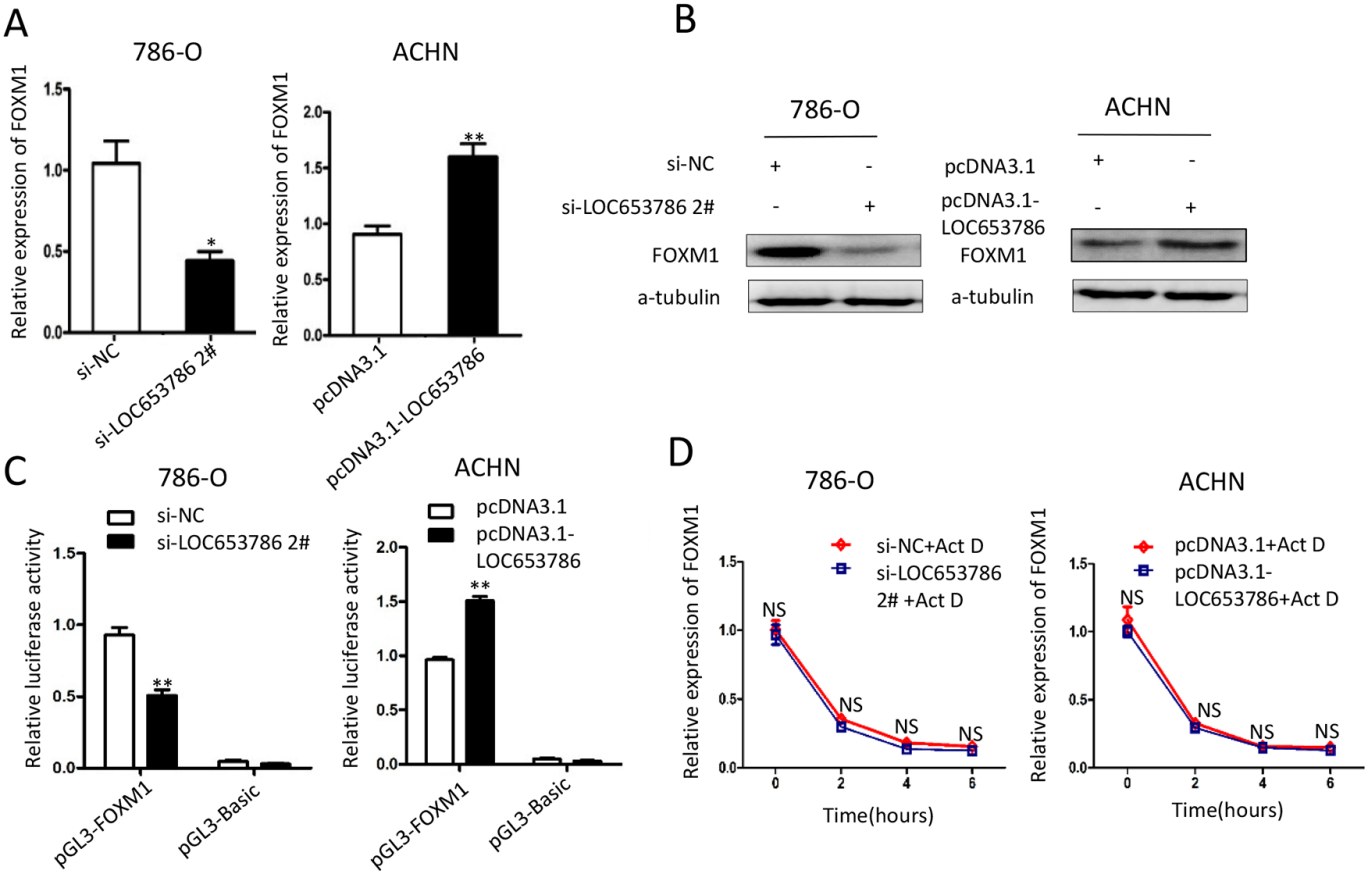
LOC653786 upregulates FOXM1 via enhancing its promoter transcriptional activity **(A, B)** 786-O and ACHN cells were separately transfected with the indicated siRNAs and expression plasmids for 48 h, then the mRNA and protein levels of FOXM1 were measured by qPCR (A) and Western blot (B). **(C)** 786-O and ACHN cells were cotransfected with pGL3-FOXM1 (or control vector pGL3-basic), pRL-TK and the indicated siRNAs or expression plasmids for 48 h, and then the firefly and renilla luciferase activities were assayed with the dual-luciferase reporter system. The ratio of firefly luminescence to renilla luminescence for each well was calculated, and the ratio of test sample was normalized against that of control. **(D)** After transfected as in (A, B), the RCC cells were treated with actinomycin D (Act D, 10μg/ml) for the indicated times. Then the mRNA level of FOXM1 was detected by qPCR. ^*^P<0.05, ^**^P<0.01, NS: no significance.

It has been reported that FOXM1 plays an essential role in promoting cell proliferation and cell cycle progression, and its two direct target genes cyclin B1 and cyclin D1 are key factors in regulating cell cycle progression. To explore whether LOC653786 could influence FOXM1 expression, we detected the mRNA and protein levels of FOXM1. As shown in Figure 4A and 4B, knockdown of LOC653786 in RCC cells markedly reduced FOXM1 expression at transcriptional and translational levels, while overexpression of LOC653786 dramatically increased the expression of FOXM1 at mRNA and protein levels. Moreover, the dual-luciferase reporter assay revealed that overexpression of LOC653786 significantly enhanced the transcriptional activity of *FOXM1* gene promoter, while knockdown of LOC653786 remarkably reduced *FOXM1* promoter activity (Figure 4C). Additionally, the actinomycin D (Act D) assay showed that the degradation of FOXM1 mRNA was not affected by silencing or overexpression of LOC653786 (Figure 4D), indicating that LOC653786 upregulates FOXM1 not through increasing its mRNA stability. The data above demonstrate that LOC653786 elevates the expression of FOXM1 via enhancing its promoter transcriptional activity.

### LOC653786 promotes RCC cell growth and cell cycle progression through upregulating FOXM1

**Figure 5:**
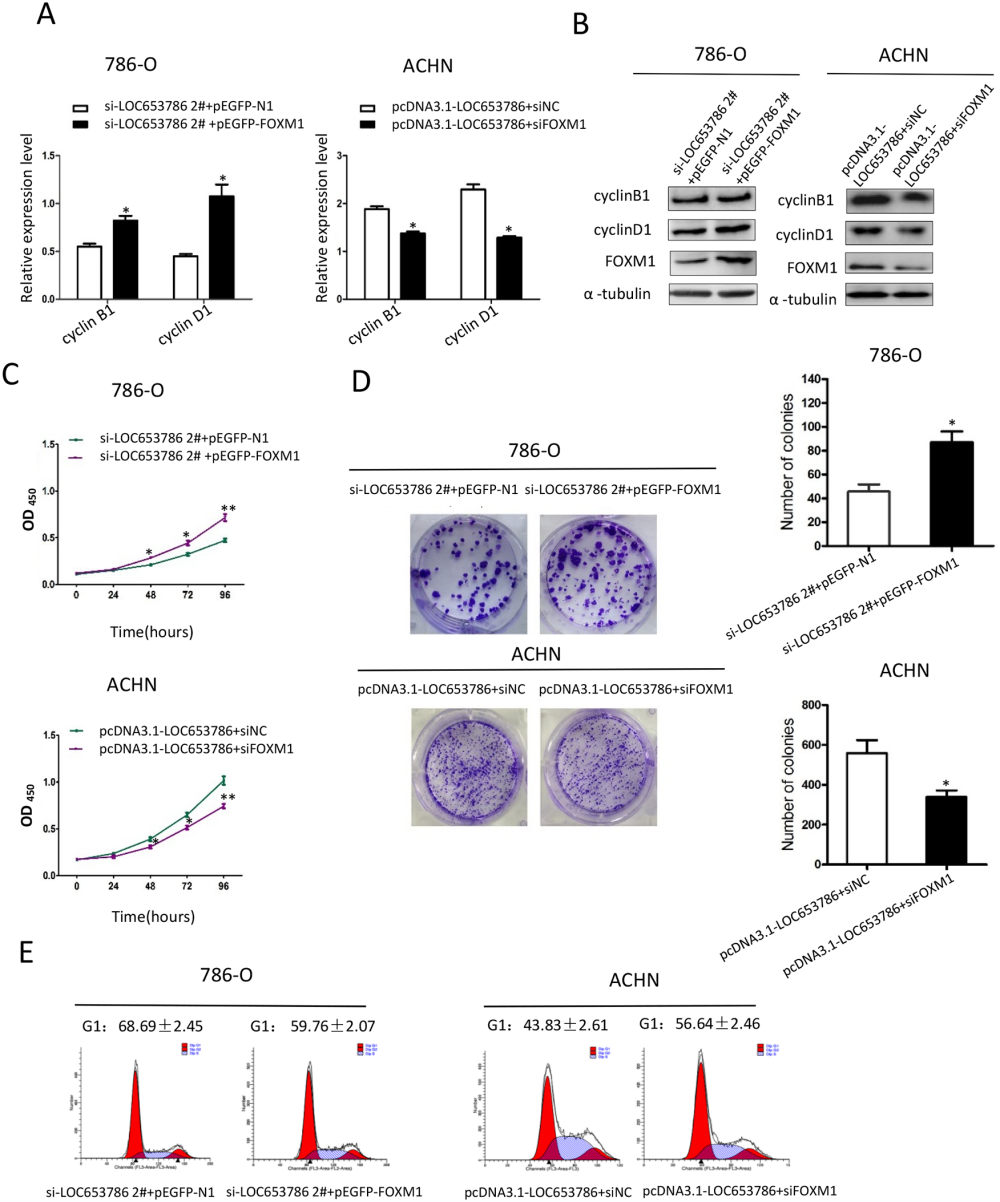
LOC653786 promotes RCC cell growth and cell cycle progression through elevating FOXM1 **(A–E)** 786-O and ACHN cells were separately cotransfected with the indicated siRNAs and expression plasmids for 48 h, and then the indicated target molecules were examined by qPCR (A) and Western blot (B), the cell viability was determined by CCK-8 assay(C), the colony-forming ability was measured by colony formation assay (D), and thecell cycle was analyzed by flow cytometry (E). ^*^P<0.05, ^**^P<0.01.

As shown in Figure 5, silencing of LOC653786 in RCC cells suppressed the expression of cyclin B1 and cyclin D1, decreased the cell viability and colony formation, and led to a cell cycle arrest at G1 phase. All the LOC653786 silencing-triggered effects above were dramatically attenuated by overexpression of FOXM1. Meanwhile, overexpression of LOC653786 in RCC cells increased the expression of cyclin B1 and cyclin D1, enhanced the cell survival and colony formation, and accelerated cell cycle progression. All the LOC653786 overexpression-induced effects above were markedly alleviated by silencing of FOXM1. Additionally, qPCR assays clarified that both the overexpression of FOXM1 in 786-O cells (Supplementery Figure 2A) and the silencing of FOXM1 in ACHN cells (Supplementery Figure 2B) were efficient. Collectively, these results indicate that LOC653786 promotes RCC cell growth and cell cycle progression via elevating FOXM1.

### Knockdown of LOC653786 represses RCC xenograft growth and FOXM1 expression *in vivo*

**Figure 6:**
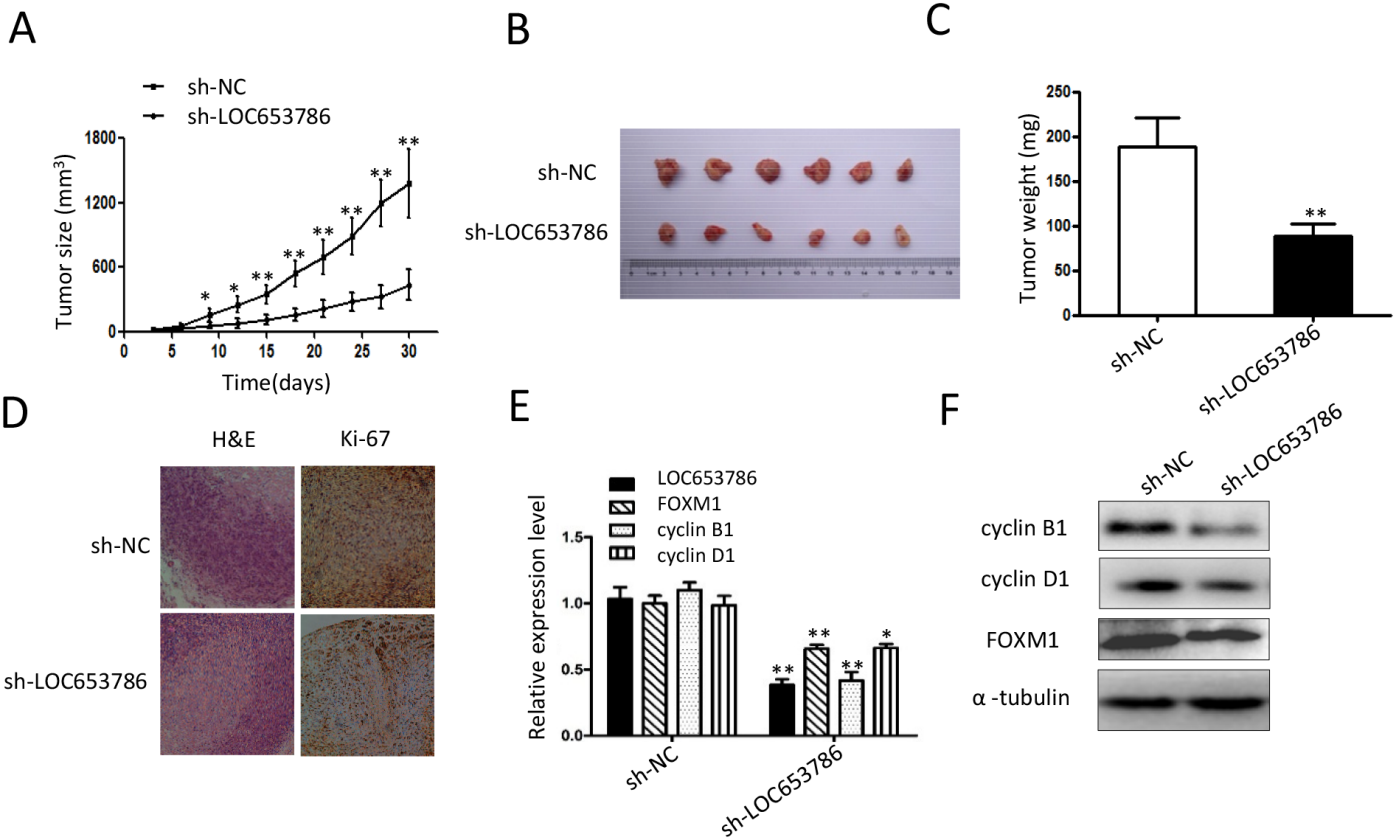
Knockdown of LOC653786 represses RCC xenograft growth and FOXM1 expression *in vivo* **(A–F)** Six-week-old male nude mice were randomly divided into two groups (n=6 per group). Then sh-LOC653786 cells (786-O cells with LOC653786 stable knockdown) and sh-NC cells (786-O cells with stable expression of shNC) were separately injected subcutaneously into the right flanks of mice (5×10^6^ cells per mouse). Subsequently, the xenograft tumor size was monitored every 3 days, and the tumor volume was calculated using the formula ‘volume = width^2^×length×1/2’ (A). Thirty days later, the mice were sacrificed, and the RCC xenografts were excised for photographing (B) and weighting (C). The level of Ki-67 in the xenograft tumors was detected by IHC staining (D), and the indicated molecules were examined by qPCR (E) and Western blot (F). ^*^P< 0.05, ^**^P< 0.01.

As shown in Figures 6A-6C, knockdown of LOC653786 markedly decreased the size and weight of RCC xenografts in nude mice. Immunohistochemisry (IHC) staining revealed that the cell proliferation marker Ki-67 was dramatically reduced in LOC653786-silencing RCC xenografts (Figure 6D). qPCR (Figure 6E) and Western blot (Figure 6F) assays showed that FOXM1 and its downstream targets cyclin B1 and cyclin D1 were notably downregulated in LOC653786-silencing RCC xenografts. In brief, the tumor-bearing experiments in nude mice indicate that knockdown of LOC653786 suppresses RCC xenograft growth and FOXM1 expression *in vivo*.

## DISCUSSION

RCC is one of the most common malignancies, and ccRCC represents the majority of RCC. ccRCC patients can show different clinical manifestation and treatment response because of the tumor heterogeneity in histology, oncogenicity and molecular alterations in tumor suppressor genes [24]. Furthermore, previous reports have shown that the mutations on Von Hippel-Lindau (*VHL*), Polybromo-1 (*PBRM1*) and BRCA1 associated protein-1 (*BAP1*) are the most common somatic mutations in ccRCC which induces the occurrence and development of ccRCC [25]. In this study, we found that LOC653786 was upregulated in ccRCC tissues compared to normal tissues by analyzing TCGA data, but it is unclear whether the higher level of LOC653786 in ccRCC tissues was associated with the mutations of *VHL, BAP1* and *PBRM1*. Analysis of the somatic mutation data from Broad GDAC Firehose (http://gdac.broadinstitute.org/) showed that there was no correlation between the expression of LOC653786 and the somatic mutations of *VHL*, *BAP1* and *PBRM1* in ccRCC tissues (Supplementery Figure 3), suggesting that the somatic mutations of the above three genes may not account for the upregulation of LOC653786. So it needs further study to explore the mechanism of LOC653786 elevation in ccRCC tissues.

LncRNA, an emerging class of ncRNA, is involved in multiple cancers including RCC. Malouf et al. have shown that 1934 lncRNAs are differentially expressed in ccRCC tissues compared to normal tissues, which are categorized into four lncRNA subclasses [26]. In addition, by using bioinformatics analysis, Blondeau et al. predicted that there were 1308 dysregulated lncRNAs in ccRCC tissues compared with normal tissues [27]. Moreover, they detected 13 representative lncRNAs in ccRCC and normal tissues with qPCR and validated that 11 lncRNAs were aberrantly expressed in ccRCC tissues [27]. Interestingly, in this study, we found that LOC653786 was included in neither the above four lncRNA subclasses nor 11 lncRNAs. Namely, LOC653786 is a novel ccRCC-associated lncRNA. Taken together, the previous reports and our data suggest that multiple lncRNAs including LOC653786 may play important roles in ccRCC.

FOXM1, an essential transcriptional factor, is involved in the promotion of cell proliferation, cell cycle progression and so on [28–30]. Importantly, a large number of studies have demonstrated that FOXM1 has a strong correlation with divergent kinds of human cancers including RCC [31–34]. It has been reported that FOXM1 enhances cancer cell growth through upregulating the cell cycle-related genes cyclin B1 and cyclin D1 [31, 32, 35]. In the present study, we revealed that LOC653786 promoted growth and cell cycle progression of RCC cells via elevating FOXM1 and its downstream targets cyclin B1 and cyclin D1, indicating that the pathway ‘LOC653786/FOXM1’ is a novel mechanism by which LOC653786 accelerates RCC cell growth and cell cycle progression.

Previous reports have summarized the myriad functions of lncRNAs into four archetypes of molecular mechanisms including signals [36, 37], decoys [38, 39], guides [40, 41] and scaffolds [42–45]. In this study, we found that the lncRNA LOC653786 upregulated FOXM1 by enhancing its promoter transcriptional activity, but it remains unknown which mechanism above is involved in the LOC653786-induced enhancement of *FOXM1* promoter activity. Therefore, it needs more studies to explore the detailed mechanism.

In conclusion, in the present study, we for the first time demonstrated that LOC653786 was highly expressed in RCC tissues and cell lines, and this lncRNA promoted growth and cell cycle progression of RCC cells via upregulating FOXM1. The *in vivo* experiments in nude mice showed that knockdown of LOC653786 dramatically repressed RCC xenograft growth and FOXM1 expression. These findings reveal that the ‘LOC653786/FOXM1’ pathway accelerates RCC cell growth, suggesting that this pathway may serve as a novel target for RCC treatment.

## MATERIALS AND METHODS

### Patients and specimens

Fifty pairs of ccRCC tissues and the corresponding adjacent non-tumor tissues were collected from Xinqiao Hospital, Third Military Medical University, from June 2015 to Mar. 2016. All the ccRCC patients were diagnosed by histopathological examination. The study was approved by the ethical committee of Third Military Medical University (Chongqing, China) and the written informed consent was obtained from all patients.

### TCGA data

ccRCC RNA-Seq data and their related clinical data were obtained from TCGA database (http://cancergenome.nih.gov/). The somatic mutation data were downloaded from The Broad GDAC Firehose (http://gdac.broadinstitute.org/).

### Cell culture

ACHN, 786-O, Caki-2 and HK-2 cell lines were purchased from Cell Bank of Chinese Academy of Sciences (Shanghai, China), and separately cultured in MEM (Gibco, Carlsbad, CA, USA), RPMI 1640 (Gibco), McCoy’s 5A (Gibco) and DMEM/F-12 (1:1) (Gibco). All the cells were supplied with 10% fetal bovine serum (FBS, Gibco) and incubated at 37°C in a 5% CO_2_ humid incubator.

### RNA extraction and qPCR

Total RNA was extracted from cells or frozen tissues with Trizol reagent (Takara, Dalian, China), and reverse transcribed to cDNA using PrimeScript™ RT reagent Kit with gDNA Eraser (Takara) according to the manufacturer’s instructions. qPCR was performed using the Power SYBR Green Master Mix Kit (Thermo Fisher Scientific Inc, Waltham, MA, USA). The expression of target genes was normalized against the internal control β-actin by using 2^-ΔΔCt^ method. The sequences of primers used for qPCR were listed in Supplementary Table 1.

### RNA FISH

RNA FISH Kit was from RiboBio (Guangzhou, China). The RNA FISH probe mix for LOC653786, 18S or U6 was synthesized and labeled with Cy3 by RiboBio. RNA FISH was performed as previously described [46]. The 6-diamidino-2-phenylindole (DAPI, RiboBio) was used for nuclei counterstaining, and the high resolution images were captured with a laser scanning confocal microscope (ZEISS, Jena, Germany).

### Plasmid construction

The DNA fragment encoding lncRNA LOC653786 was chemically synthesized by Sangon (Shanghai, China) and inserted into the expression vector pcDNA-3.1 (Invitrogen, Carlsbad, CA, USA), and the resulting recombinant plasmid was named as pcDNA3.1-LOC653786. The expression vector of FOXM1 (pEGFP-FOXM1) and the negative control vector (pEGFP-N1) were purchased from Genepharma (Shanghai, China). The promoter region of *FOXM1* gene (-1975 to +21) was chemically synthesized (Sangon) and inserted into pGL3-Basic vector (Promega, Madison, WI, USA), and the resulting recombinant plasmid was named as pGL3-FOXM1. Monitor plasmid pRL-TK was from Promega.

### Transfection assay

The siRNAs for LOC653786 (or FOXM1) and control siRNA were from Genepharma. The siRNA sequences were listed in Supplementary Table 2. Transfections were performed using Lipofectamine 3000 (Invitrogen) according to the manufacturer’s instructions. Briefly, RCC cells were seeded into culture plates and cultured overnight. Then siRNA and Lipofectamine 3000 were separately diluted with Opti-MEM (Gibco). Meanwhile, plasmid and P3000 were diluted together with Opti-MEM. Subsequently, the diluted siRNA (or plasmid) and the diluted Lipofectamine 3000 were mixed together, and the mixtures were incubated for 15 min at room temperature. Next, the cell culture medium was removed and the fresh culture medium with corresponding mixture was added into culture plates. After incubation for the appropriate time, the subsequent experiments were performed.

### Cell viability assay

The cell viability was assayed using a Cell Counting Kit-8 (CCK-8) from Dojindo (Kumamoto, Japan) as previously described [47]. Briefly, the cells were seeded into 96-well plates and cultured overnight, followed by the different treatments for the indicated times. Then 10 μl CCK-8 solution was added to each well. After incubation at 37°C for 1h, the OD values at 450 nm were determined with a microplate reader (Tecan, Austria) and normalized against that of the corresponding control.

### Colony formation assay

A certain number of transfected cells were placed on 6-well plates and maintained in an appropriate medium containing 10% FBS for 12 days, during which the medium was replaced every 3 days. Subsequently, the colonies were fixed overnight with 4% paraformaldehyde solution, and then stained by 0.1% crystal violet (Beyotime, Shanghai, China) for 1 h. Finally, the visible colonies were photographed and counted manually.

### Flow cytometry assay

The cells with different treatments were trypsinized and collected into centrifuge tubes. Next, the cells were washed with phosphate-buffered saline (PBS) for 2 times, and then fixed with pre-cooling 70% ethanol overnight at 4°C. After washing three times with cold PBS, the cells were treated with RNase A (50 μg/ml) (Sigma, MO, USA) in PBS at 37°C for 30 min. Thereafter, the cells were incubated with propidium iodide (PI, 50 μg/ml) (BD Biosciences, CA, USA) at darkness for 15 min, and then the cell cycle was analyzed by a MoFlo XDP flow cytometer (Beckman Coulter, CA, USA).

### Western blot

The proteins were extracted and the concentrations were measured with a BCA protein assay Kit (Beyotime). Then Western blot assay was performed to detect the specific protein as previously described [48]. The target protein levels were normalized against that of the control α-tubulin. The primary antibodies anti-FOXM1 and anti-α-tubulin were purchased from Abcam corporation (Cambridge, MA, USA), and anti-cyclin B1 and anti-cyclin D1 were bought from Cell Signaling Technology (Boston, MA, USA). The second antibodies horseradish peroxidase-labeled goat anti-rabbit IgG and goat anti-mouse IgG were from Zhongshan Biotechnologies (Beijing, China). The ECL chemiluminescence kit was from Beyotime.

### Dual-luciferase reporter assay

After grown to 70%-80% confluence in culture plates, the cells were cotransfected with pGL3-FOXM1, pRL-TK and the indicated siRNAs or expression vectors. Forty-eight hours later, the cells were lysed, and the firefly and renilla luciferase activities were measured using the Dual-Luciferase Reporter System (Promega) according to the manufacturer’s instructions. The ratio of firefly luminescence to renilla luminescence for each well was calculated, and the ratio of test sample was normalized against that of the control.

### Establishment of stable-expressing cell lines

The DNA fragments encoding LOC653786 shRNA (shLOC653786) and scramble shRNA (shNC) were synthesized by Genepharma, and separately inserted into the vector pGLV3/H1/GFP/Puro (Genepharma), and the resulting plasmids were named as pGLV3/H1/GFP/Puro/sh-LOC653786 and pGLV3/H1/GFP/Puro/sh-NC. Then the packaged lentivirus containing above two plasmids were used to infect 786-O cells at a multiplicity of infection (MOI) of 10, followed by screening with puromycin (Solarbio Life Sciences, Beijing, China). The resulting 786-O cells with stable knockdown of LOC653786 was named as sh-LOC653786 cell line, and the control 786-O cells with stable expression of shNC was named as sh-NC cell line. The DNA sequences separately encoding shLOC653786 and shNC were 5′-GCTCGCCTGTCTACTAACTAA-3′ and 5′-GCTCGCCTGTCTACTAACTAA-3′.

### RCC Xenograft experiments *in vivo*

Six-week-old male nude mice were obtained from Beijing Huafukang Bioscience (Beijing, China), housed and cared for under the regulations of the guidelines of the Animal Care and Ethics Committee of Third Military Medical University (Chongqing, China). Twelve nude mice were randomly divided into two groups (n=6 per group). Subsequently, the above two stable-expressing cell lines (sh-LOC653786 and sh-NC) were separately injected subcutaneously into the right flanks of mice (5×10^6^ cells per mouse). The xenograft tumor size was monitored every 3 days, and the tumor volume was calculated using the formula ‘volume = width^2^×length×1/2’. Thirty days later, the mice were sacrificed and the xenograft tumors were harvested. The corresponding target mRNAs and proteins in the xenograft tumors were separately examined by qPCR, IHC staining and Western blot.

### IHC staining

IHC staining was performed as previously described [12]. After fixed with 4% paraformaldehyde overnight, the xenograft tumor tissues were embedded in paraffin and sliced at 5 μm thickness. Then the tumor sections were immunostained with Histostain-Plus Kits from Beijing Zhongshan Biotechnologies according to the manufacture instructions. Briefly, the sections were treated with 3% H_2_O_2_ to block endogenous peroxidase, followed by the incubation with 0.1% trypsin for antigen demasking. Subsequently, the sections were blocked with 5% goat serum (Zhongshan Biotechnologies) for 30 min at 37°C, and then incubated with the Ki-67 antibody (Abcam) overnight at 4°C. After warming and washing, the sections were incubated with the secondary antibody horseradish peroxidase-labeled goat anti-rabbit IgG for 30 min at 37°C. Finally, the sections were counterstained with Alcian blue and photographed under a microscope.

### Statistical analysis

All data were displayed as mean ± SD (standard deviation). The difference between 2 groups was analyzed with Student *t* test. All statistical analyses were done with Statistical Package for the Social Sciences (SPSS), version 17.0 (SPSS Inc., IL, USA). *P* < 0.05 was considered statistically significant.

## SUPPLEMENTARY MATERIALS FIGURES AND TABLES


