# Supplementary material for: LncRNA LOC653786 promotes growth of RCC cells via upregulating FOXM1
Source: Oncotarget. 2018 Jan 8;9(15):12101–11. doi: 10.18632/oncotarget.24027 (PMC5844731; doi:10.18632/oncotarget.24027)
Supplement: Supplementary file 1 [file oncotarget-09-12101-s001.pdf]

LncRNA LOC653786 promotes growth of RCC cells via upregulating FOXM1

SUPPLEMENTARY MATERIALS

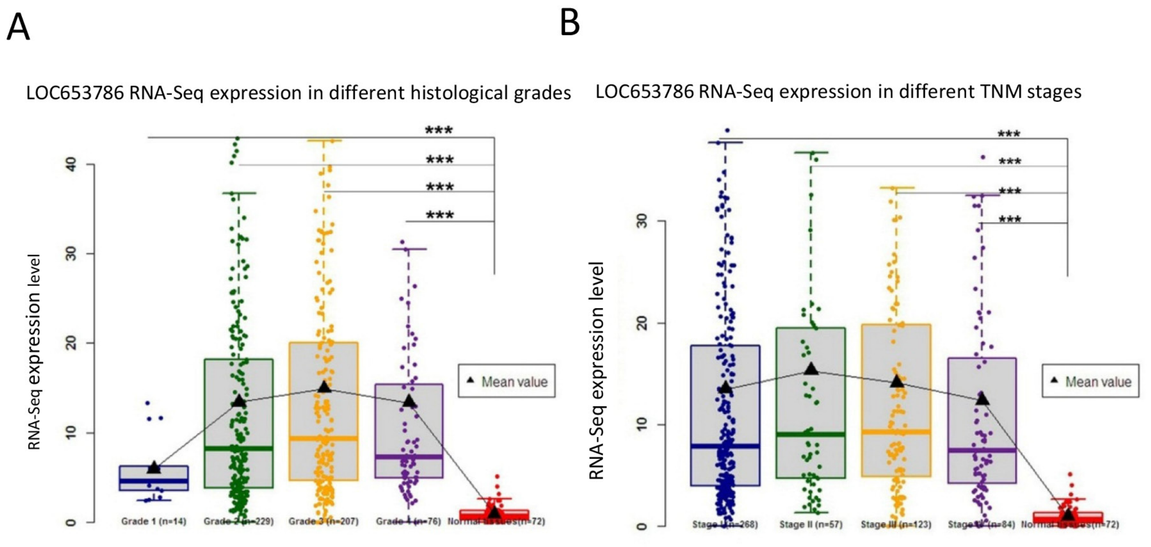

Supplementary Figure 1: LOC653786 is highly expressed in RCC tissues in different histological grades and TNM stages. Analysis of LOC653786 RNA-Seq expression in different histological grades **(A)** and TNM stages **(B)** of ccRCC tissues and normal tissues using TCGA data (http://cancergenome.nih.gov/). $^{***}P < 0.001$.

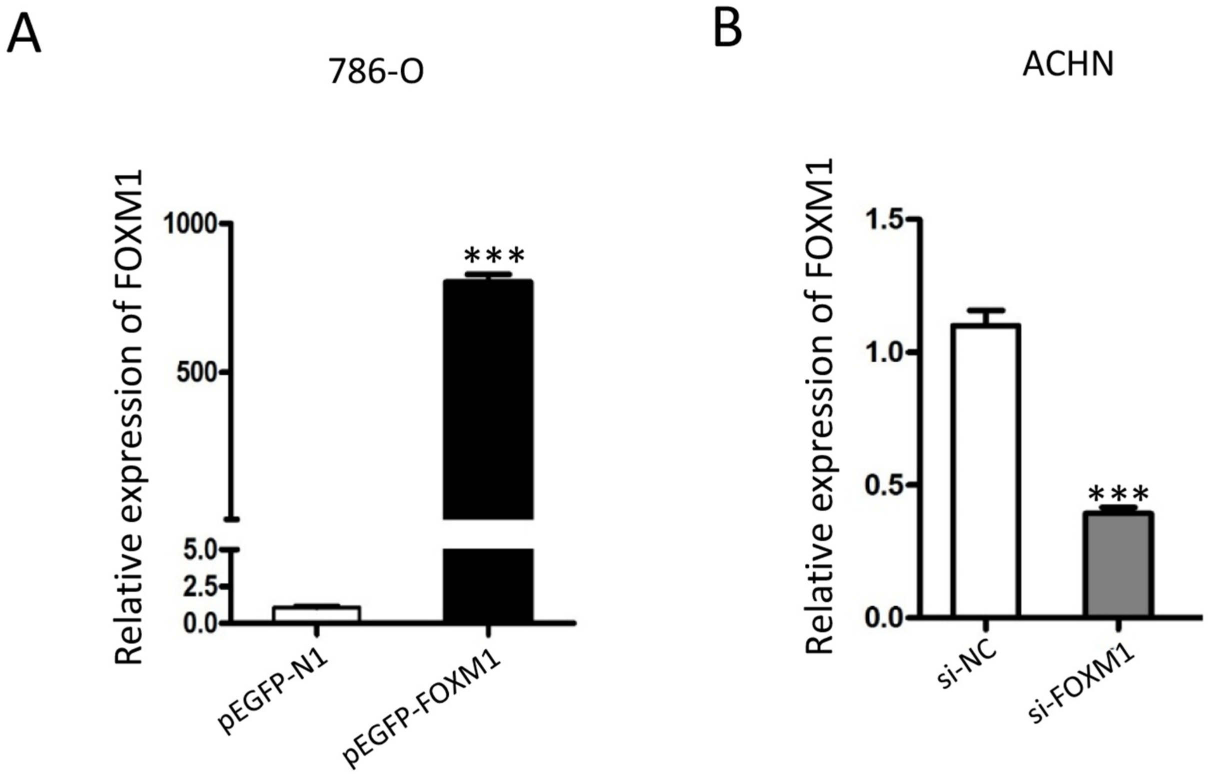

Supplementary Figure 2: Both the overexpression and silencing of FOXM1 in RCC cells are efficient. **(A)** 786-O cells were transfected with FOXM1 expression plasmid pEGFP-FOXM1 or control vector pEGFP-N1 for 48 h, and then the level of FOXM1 mRNA was examined by qPCR. **(B)** ACHN cellswere transfected with FOXM1 siRNA (si-FOXM1) or control siRNA (si-NC) for 48 h, and then the level of FOXM1 mRNA was detected by qPCR. $^{***}P < 0.001$.

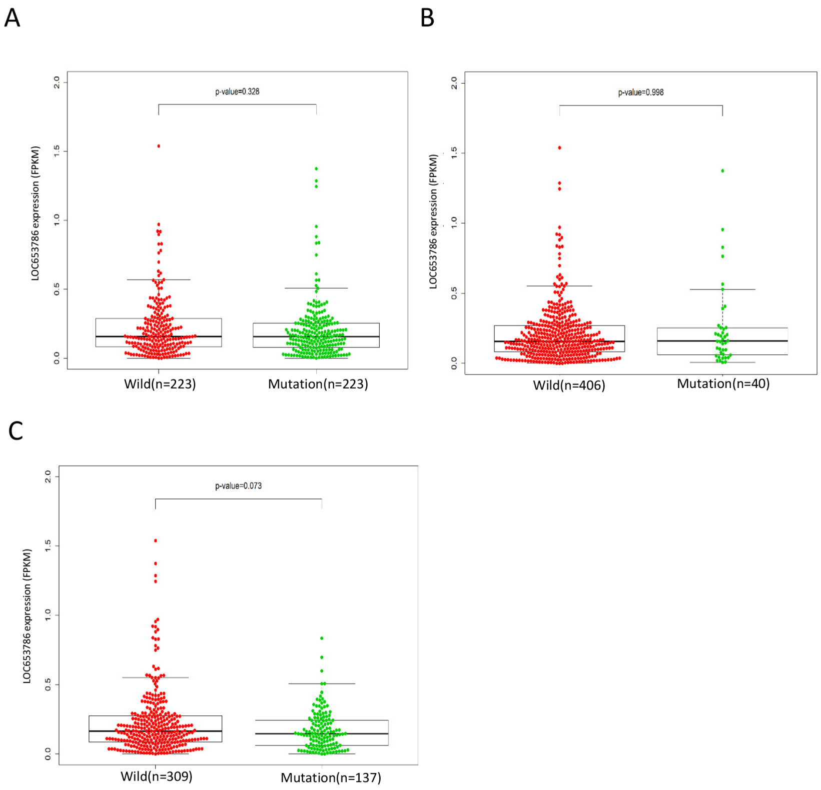

Supplementary Figure 3: There is no correlation between LOC653786 expression and somatic mutations of *VHL*, *BAP1* and *PBRM1* in RCC tissues. (A-C) Analysis of the difference of LOC653786expression between *VHL* wild-type and mutation groups **(**A**)**, *BAP1* wild-type and mutation groups **(**B**)**, and *PBRM1* wild-type and mutation groups in ccRCC tissues using TCGA data (http://cancergenome.nih.gov/ and http://gdac.broadinstitute.org/).

Supplementary Table 1: The primer sets used in qPCR

Targets	Forward primers (5′→3′)	Reverse primers (5′→3′)
hFOXM1	AGTAGTGGGCCCAACAAATTCAT	CTTTTGGCATCATAGCTGGTTTG
hcyclin B1	TCTGGATAATGGTGAATGGACA	CGATGTGGCATACTTGTTCTTG
hcyclin D1	CGCCCCACCCCTCCAG	CCGCCCAGACCCTCAGACT
hLOC653786	GGAAAAGGCAACAGATGTCCA	GGAAGCTGCTTGCAGAAGGAT
hβ-actin	GTGAAGGTGACAGCAGTCGGTT	GAAGTGGGGTGGCTTTTAGGA

h: human.

Supplementary Table 2: The sequences of siRNAs

siRNAs	Sequences (5′→3′)
siFOXM1 (565)	GUGGGCCCAACAAAUUCAUTT
siLOC653786-1# (1141)	GGGACCAUGUGAUUGACAUTT
siLOC653786-2# (603)	GCUCUUCACUGUAGGCAUUTT
siNC	UUCUCCGAACGUGUCACGUTT
